# {1,2-Bis[(3,5-dimethyl-1*H*-pyrazol-1-yl-κ*N* ^2^)meth­yl]benzene}­dichloridozinc(II)

**DOI:** 10.1107/S1600536811046368

**Published:** 2011-11-09

**Authors:** Ilia A. Guzei, Lara C. Spencer, Asheena Budhai, James Darkwa

**Affiliations:** a Department of Chemistry, University of Wisconsin-Madison, 1101 University Ave, Madison, WI 53706, USA; b Department of Chemistry, University of Johannesburg, Auckland Park Kingsway Campus, Johannesburg 2006, South Africa

## Abstract

The title zinc complex, [ZnCl_2_(C_18_H_22_N_4_)], contains a bidentate 1,2-bis(3,5-dimethyl-1*H*-pyrazol-1-ylmeth­yl)benz­ene ligand that binds to the zinc atom, forming a nine-membered metallocyclic ring. The geometry about the Zn atom is distorted tetra­hedral, with the largest deviation observed in the magnitude of the Cl—Zn—Cl angle. Similar distortions are observed in the cobalt analogue and related zinc compounds containing metallocyclic rings with more than six members. The copper analogue exhibits a more severe distortion of the metal coordination sphere than is observed in the title compound.

## Related literature

For the coordination modes of poly(pyrazol-1-ylmeth­yl)benzene see: Hartshorn & Steel (1995 ▶, 1997 ▶, 1998 ▶); Guerrero *et al.* (2002 ▶). For 1,2-bis(3,5-dimethyl-1*H*-pyrazol-1-ylmeth­yl)benzene complexes with palladium in square-planar coord­ination, see: Motsoane *et al.* (2007 ▶). For the cobalt and copper analogues, see: Chang *et al.* (1994 ▶). Discussion of the effect of the size of metallocyclic rings on the distortion of tetra­hedral dipyrazole dizinc complexes can be found in Guzei *et al.* (2011 ▶). Related structures were found in the Cambridge Structural Database (Allen, 2002 ▶). Bond lengths and angles were confirmed to be typical by a *Mogul* structural check (Bruno *et al.*, 2002 ▶).
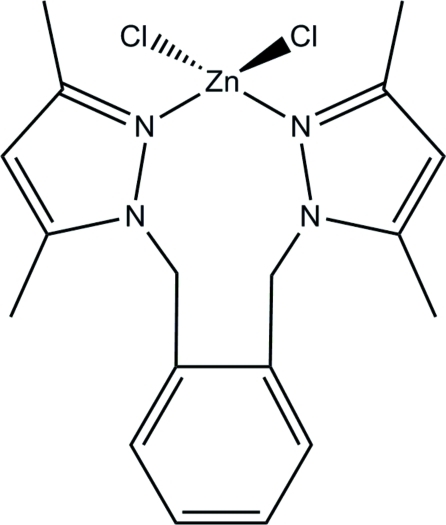


## Experimental

### 

#### Crystal data

[ZnCl_2_(C_18_H_22_N_4_)]
*M* *_r_* = 430.67
Triclinic, 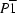

*a* = 9.0830 (8) Å
*b* = 10.6375 (9) Å
*c* = 11.9558 (10) Å
α = 111.853 (1)°
β = 95.476 (1)°
γ = 112.636 (1)°
*V* = 950.38 (14) Å^3^
*Z* = 2
Mo *K*α radiation
μ = 1.58 mm^−1^
*T* = 100 K
0.43 × 0.32 × 0.28 mm

#### Data collection

Bruker CCD-1000 area-detector diffractometer
Absorption correction: multi-scan (*SADABS*; Bruker, 2003 ▶) *T*_min_ = 0.550, *T*_max_ = 0.666
13237 measured reflections
4672 independent reflections
4403 reflections with *I* > 2σ(*I*)
*R*_int_ = 0.020

#### Refinement

*R*[*F* ^2^ > 2σ(*F* ^2^)] = 0.024
*wR*(*F* ^2^) = 0.065
*S* = 1.04
4672 reflections
230 parameters
H-atom parameters constrained
Δρ_max_ = 0.47 e Å^−3^
Δρ_min_ = −0.23 e Å^−3^

### 

Data collection: *SMART* (Bruker, 2003 ▶); cell refinement: *SAINT* (Bruker, 2003 ▶); data reduction: *SAINT*; program(s) used to solve structure: *SHELXTL* (Sheldrick, 2008 ▶); program(s) used to refine structure: *SHELXTL* and *FCF_filter* (Guzei, 2007 ▶); molecular graphics: *SHELXTL* and *DIAMOND* (Brandenburg, 1999 ▶); software used to prepare material for publication: *SHELXTL*, *publCIF* (Westrip, 2010 ▶) and *modiCIFer* (Guzei, 2007 ▶).

## Supplementary Material

Crystal structure: contains datablock(s) global, I. DOI: 10.1107/S1600536811046368/lr2035sup1.cif

Structure factors: contains datablock(s) I. DOI: 10.1107/S1600536811046368/lr2035Isup2.hkl

Additional supplementary materials:  crystallographic information; 3D view; checkCIF report

## Figures and Tables

**Table:** 

Zn1—N1	2.0323 (11)
Zn1—N4	2.0512 (11)
Zn1—Cl2	2.2145 (4)
Zn1—Cl1	2.2526 (4)

**Table:** 

N1—Zn1—N4	111.72 (4)
N1—Zn1—Cl2	115.14 (3)
N4—Zn1—Cl2	104.72 (3)
N1—Zn1—Cl1	103.37 (3)
N4—Zn1—Cl1	106.19 (3)
Cl2—Zn1—Cl1	115.538 (13)

**Fig. 1.**
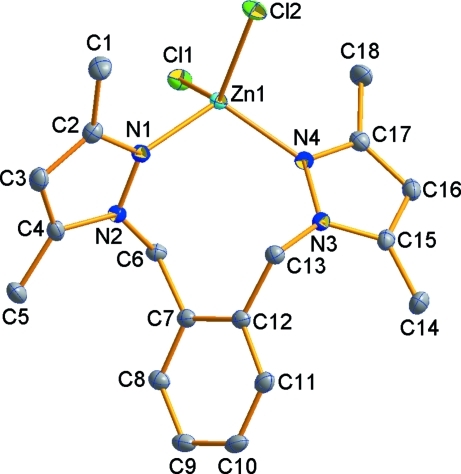
Molecular structure of (I) (Brandenburg, 1999). The thermal ellipsoids are shown at 50% probability level. All hydrogen atoms were omitted for clarity.

## supplementary crystallographic information

### Comment

Poly(pyrazol-1-ylmethyl)benzene exhibits several coordination modes depending on the positions of the pyrazolyl unit on the benzene ring (Hartshorn & Steel, 1995, 1997, 1998; Guerrero *et al.* 2002). There are two types of coordination for the 1,2-bis(pyrazol-1-ylmethyl)benzene analogue. For square planar complexes 1,2-bis(pyrazol-1-ylmethyl)benzene behaves as a monodentate ligand as observed for palladium (Motsoane *et al.*, 2007), whereas for tetrahedral complexes the ligand is bidentate binding to the metal center through nitrogen atoms on each of the pyrazole groups.

In the title complex (I) the bis-pyrazolyl ligand binds to the zinc center to form a nine-membered metallocycle. The dependence of the magnitude of the N—Zn—N angle on the size of the metallocycle was discussed by Guzei *et al.* (2011) for 2-(3,5-dimethyl-pyrazol-1-yl)-ethylamine zinc(II) chloride (II). In (I) the N—Zn—N angle (111.72 (4)°) is much closer to the ideal tetrahedral value because the size of the ring (nine atoms) exceeds 6, whereas in (II) the metallocycle is six-membered and more sterically constrained, which leads to a smaller angle of 96.88 (6)°. The Zn center in (I) possesses a distorted tetrahedral geometry; the dihedral angle between the planes defined by atoms Zn1, N1, N4 and Zn1, Cl1, Cl2 spans 85.91 (3)°, which is in good agreement with the corresponding angle of 86.77 (4)° in (II). The geometrical distortion of the Zn coordination sphere can be compared to those in the Co and Cu analogues ((III) and (IV), Chang *et al.*, 1994). The Cu analogue is substantially more distorted as revealed by the following values of the Cl—M—Cl angle, N—M—N angle, and a range of the N—M—Cl angles. These values for (I), (III) and (IV), correspondingly are 115.538 (13), 111.72 (4), 103.37 (3)–115.14 (3)°; 115.50 (3), 110.62 (8), 102.56 (6)–112.71 (6)°; 133.4 (7),141 (7), 95.01 (13)–100.68 (12)°. It is noteworthy that the overall molecular geometry of (I) approximately conforms to C_2_-symmetry, whereas geometries of (III) and (IV) are essentially C_S_-symmetrical.

The distortion in (I) is noticeably smaller than in relevant compounds in the Cambridge Structural Database (Allen, 2002; Guzei *et al.* 2011). A structural check of (I) in *Mogul* confirmed its other geometrical parameters to be typical (Bruno *et al.* 2002).

### Experimental

A mixture of solid 1,2-bis(pyrazol-1-ylmethyl)benzene (0.27 g, 0.92 mmol) and anhydrous ZnCl_2_ (0.120 g, 0.92 mmol) was dissolved in methanol (20 ml), and the resulting solution stirred at room temperature for 12 h. The solvent was removed *in vacuo* and the residue recrystallized from dichloromethane and hexane. Yield: 0.25 g (64%).

### Refinement

All H-atoms attached to carbon atoms were placed in idealized locations and refined as riding with appropriate thermal displacement coefficients *U*_iso_(H) = 1.5 times *U*_eq_(bearing atom) for methyl H atoms and *U*_iso_(H) = 1.2 times *U*_eq_(bearing atom) for all other H atoms. Default effective *X*—H distances for T = -173.0 ° C C(*sp* 3)–2H=0.99, C(*sp* 3)–3H=0.98, C(*sp* 2)–H=0.95.

### Crystal data

**Table:**

[ZnCl_2_(C_18_H_22_N_4_)]	*Z* = 2
*M**_r_* = 430.67	*F*(000) = 444
Triclinic, *P*1	*D*_x_ = 1.505 Mg m^−^^3^
Hall symbol: -P 1	Mo *K*α radiation, λ = 0.71073 Å
*a* = 9.0830 (8) Å	Cell parameters from 9448 reflections
*b* = 10.6375 (9) Å	θ = 2.3–28.3°
*c* = 11.9558 (10) Å	µ = 1.58 mm^−^^1^
α = 111.853 (1)°	*T* = 100 K
β = 95.476 (1)°	Block, colourless
γ = 112.636 (1)°	0.43 × 0.32 × 0.28 mm
*V* = 950.38 (14) Å^3^	

### Data collection

**Table:**

Bruker CCD-1000 area-detector diffractometer	4672 independent reflections
Radiation source: fine-focus sealed tube	4403 reflections with *I* > 2σ(*I*)
graphite	*R*_int_ = 0.020
0.30° ω scans	θ_max_ = 28.3°, θ_min_ = 2.3°
Absorption correction: multi-scan (*SADABS*; Bruker, 2003)	*h* = −12→12
*T*_min_ = 0.550, *T*_max_ = 0.666	*k* = −14→14
13237 measured reflections	*l* = −15→15

### Refinement

**Table:**

Refinement on *F*^2^	Primary atom site location: structure-invariant direct methods
Least-squares matrix: full	Secondary atom site location: difference Fourier map
*R*[*F*^2^ > 2σ(*F*^2^)] = 0.024	Hydrogen site location: inferred from neighbouring sites
*wR*(*F*^2^) = 0.065	H-atom parameters constrained
*S* = 1.04	*w* = 1/[σ^2^(*F*_o_^2^) + (0.0389*P*)^2^ + 0.3716*P*] where *P* = (*F*_o_^2^ + 2*F*_c_^2^)/3
4672 reflections	(Δ/σ)_max_ = 0.001
230 parameters	Δρ_max_ = 0.47 e Å^−^^3^
0 restraints	Δρ_min_ = −0.23 e Å^−^^3^

### Special details

**Table:**

Geometry. All e.s.d.'s (except the e.s.d. in the dihedral angle between two l.s. planes) are estimated using the full covariance matrix. The cell e.s.d.'s are taken into account individually in the estimation of e.s.d.'s in distances, angles and torsion angles; correlations between e.s.d.'s in cell parameters are only used when they are defined by crystal symmetry. An approximate (isotropic) treatment of cell e.s.d.'s is used for estimating e.s.d.'s involving l.s. planes.
Refinement. Refinement of *F*^2^ against ALL reflections. The weighted *R*-factor *wR* and goodness of fit *S* are based on *F*^2^, conventional *R*-factors *R* are based on *F*, with *F* set to zero for negative *F*^2^. The threshold expression of *F*^2^ > σ(*F*^2^) is used only for calculating *R*-factors(gt) *etc*. and is not relevant to the choice of reflections for refinement. *R*-factors based on *F*^2^ are statistically about twice as large as those based on *F*, and *R*- factors based on ALL data will be even larger.

### Fractional atomic coordinates and isotropic or equivalent isotropic displacement parameters (Å^2^)

**Table:**

	*x*	*y*	*z*	*U*_iso_*/*U*_eq_	
Zn1	0.447154 (17)	0.646823 (15)	0.225301 (13)	0.01268 (6)	
Cl1	0.33460 (4)	0.70562 (4)	0.38540 (3)	0.01945 (8)	
Cl2	0.36462 (4)	0.40099 (3)	0.11091 (3)	0.02033 (8)	
N1	0.39991 (13)	0.75623 (12)	0.12959 (10)	0.0141 (2)	
N2	0.45625 (13)	0.91072 (12)	0.18533 (10)	0.0134 (2)	
N3	0.83014 (13)	0.82791 (12)	0.28085 (10)	0.0142 (2)	
N4	0.69672 (13)	0.73599 (12)	0.30515 (10)	0.0150 (2)	
C1	0.19365 (18)	0.53631 (16)	−0.06690 (14)	0.0231 (3)	
H1A	0.1488	0.4774	−0.0211	0.035*	
H1B	0.2708	0.5047	−0.1071	0.035*	
H1C	0.1024	0.5183	−0.1311	0.035*	
C2	0.28349 (16)	0.70176 (15)	0.02240 (12)	0.0168 (2)	
C3	0.26618 (17)	0.82135 (16)	0.01074 (13)	0.0192 (3)	
H3	0.1924	0.8139	−0.0560	0.023*	
C4	0.37729 (16)	0.95269 (15)	0.11533 (13)	0.0164 (2)	
C5	0.40993 (18)	1.11322 (16)	0.15328 (15)	0.0232 (3)	
H5A	0.5219	1.1720	0.1503	0.035*	
H5B	0.4011	1.1574	0.2388	0.035*	
H5C	0.3282	1.1160	0.0957	0.035*	
C6	0.58545 (15)	1.01004 (14)	0.30583 (12)	0.0141 (2)	
H6A	0.6018	0.9463	0.3438	0.017*	
H6B	0.5468	1.0772	0.3633	0.017*	
C7	0.75061 (15)	1.10757 (14)	0.29404 (12)	0.0141 (2)	
C8	0.80300 (17)	1.26401 (15)	0.34196 (12)	0.0170 (2)	
H8	0.7353	1.3055	0.3812	0.020*	
C9	0.95267 (17)	1.36036 (15)	0.33336 (13)	0.0194 (3)	
H9	0.9869	1.4666	0.3671	0.023*	
C10	1.05119 (16)	1.30043 (16)	0.27536 (13)	0.0198 (3)	
H10	1.1526	1.3650	0.2679	0.024*	
C11	1.00063 (16)	1.14496 (15)	0.22812 (13)	0.0177 (2)	
H11	1.0685	1.1044	0.1881	0.021*	
C12	0.85275 (15)	1.04717 (14)	0.23806 (12)	0.0145 (2)	
C13	0.80739 (16)	0.87931 (14)	0.18550 (12)	0.0146 (2)	
H13A	0.6897	0.8196	0.1348	0.017*	
H13B	0.8760	0.8575	0.1286	0.017*	
C14	1.13949 (17)	0.93722 (17)	0.33455 (15)	0.0228 (3)	
H14A	1.1667	1.0453	0.3732	0.034*	
H14B	1.1362	0.9036	0.2457	0.034*	
H14C	1.2243	0.9222	0.3773	0.034*	
C15	0.97434 (16)	0.84694 (15)	0.34583 (13)	0.0167 (2)	
C16	0.93254 (17)	0.76621 (15)	0.41487 (13)	0.0183 (3)	
H16	1.0067	0.7584	0.4705	0.022*	
C17	0.75974 (17)	0.69826 (15)	0.38680 (13)	0.0172 (2)	
C18	0.65037 (18)	0.59516 (17)	0.43404 (15)	0.0236 (3)	
H18A	0.5678	0.5006	0.3627	0.035*	
H18B	0.5936	0.6454	0.4858	0.035*	
H18C	0.7181	0.5720	0.4846	0.035*	

### Atomic displacement parameters (Å^2^)

**Table:**

	*U*^11^	*U*^22^	*U*^33^	*U*^12^	*U*^13^	*U*^23^
Zn1	0.01343 (8)	0.01059 (8)	0.01304 (9)	0.00483 (6)	0.00326 (6)	0.00506 (6)
Cl1	0.02365 (16)	0.01755 (15)	0.01855 (16)	0.00964 (13)	0.01082 (12)	0.00796 (12)
Cl2	0.02673 (17)	0.01197 (14)	0.01843 (16)	0.00796 (12)	0.00488 (12)	0.00416 (12)
N1	0.0146 (5)	0.0122 (5)	0.0141 (5)	0.0056 (4)	0.0033 (4)	0.0052 (4)
N2	0.0134 (5)	0.0122 (5)	0.0146 (5)	0.0055 (4)	0.0036 (4)	0.0063 (4)
N3	0.0129 (5)	0.0140 (5)	0.0155 (5)	0.0060 (4)	0.0040 (4)	0.0065 (4)
N4	0.0142 (5)	0.0141 (5)	0.0162 (5)	0.0054 (4)	0.0039 (4)	0.0073 (4)
C1	0.0227 (7)	0.0197 (6)	0.0180 (7)	0.0080 (5)	−0.0017 (5)	0.0033 (5)
C2	0.0153 (6)	0.0196 (6)	0.0149 (6)	0.0076 (5)	0.0043 (5)	0.0073 (5)
C3	0.0184 (6)	0.0244 (7)	0.0179 (6)	0.0108 (5)	0.0040 (5)	0.0116 (5)
C4	0.0165 (6)	0.0199 (6)	0.0198 (6)	0.0107 (5)	0.0078 (5)	0.0127 (5)
C5	0.0228 (7)	0.0203 (6)	0.0342 (8)	0.0127 (6)	0.0092 (6)	0.0165 (6)
C6	0.0156 (6)	0.0126 (5)	0.0122 (5)	0.0053 (5)	0.0039 (4)	0.0051 (5)
C7	0.0144 (5)	0.0141 (5)	0.0122 (5)	0.0048 (5)	0.0022 (4)	0.0065 (5)
C8	0.0199 (6)	0.0152 (6)	0.0146 (6)	0.0068 (5)	0.0030 (5)	0.0069 (5)
C9	0.0218 (6)	0.0143 (6)	0.0171 (6)	0.0033 (5)	0.0011 (5)	0.0080 (5)
C10	0.0150 (6)	0.0197 (6)	0.0202 (6)	0.0015 (5)	0.0016 (5)	0.0120 (5)
C11	0.0150 (6)	0.0212 (6)	0.0174 (6)	0.0068 (5)	0.0037 (5)	0.0108 (5)
C12	0.0146 (6)	0.0150 (6)	0.0132 (6)	0.0054 (5)	0.0023 (4)	0.0074 (5)
C13	0.0150 (6)	0.0153 (6)	0.0137 (6)	0.0067 (5)	0.0044 (4)	0.0068 (5)
C14	0.0147 (6)	0.0255 (7)	0.0303 (8)	0.0095 (5)	0.0061 (5)	0.0141 (6)
C15	0.0146 (6)	0.0164 (6)	0.0180 (6)	0.0086 (5)	0.0027 (5)	0.0055 (5)
C16	0.0182 (6)	0.0174 (6)	0.0184 (6)	0.0093 (5)	0.0009 (5)	0.0068 (5)
C17	0.0196 (6)	0.0154 (6)	0.0160 (6)	0.0081 (5)	0.0031 (5)	0.0066 (5)
C18	0.0230 (7)	0.0250 (7)	0.0265 (7)	0.0085 (6)	0.0040 (5)	0.0183 (6)

### Geometric parameters (Å, °)

**Table:**

Zn1—N1	2.0323 (11)	C6—H6B	0.9900
Zn1—N4	2.0512 (11)	C7—C8	1.3974 (17)
Zn1—Cl2	2.2145 (4)	C7—C12	1.4066 (18)
Zn1—Cl1	2.2526 (4)	C8—C9	1.3934 (19)
N1—C2	1.3466 (17)	C8—H8	0.9500
N1—N2	1.3707 (14)	C9—C10	1.384 (2)
N2—C4	1.3506 (16)	C9—H9	0.9500
N2—C6	1.4693 (16)	C10—C11	1.3912 (19)
N3—C15	1.3581 (16)	C10—H10	0.9500
N3—N4	1.3695 (15)	C11—C12	1.3954 (18)
N3—C13	1.4654 (16)	C11—H11	0.9500
N4—C17	1.3429 (17)	C12—C13	1.5173 (17)
C1—C2	1.4942 (19)	C13—H13A	0.9900
C1—H1A	0.9800	C13—H13B	0.9900
C1—H1B	0.9800	C14—C15	1.4889 (19)
C1—H1C	0.9800	C14—H14A	0.9800
C2—C3	1.3944 (19)	C14—H14B	0.9800
C3—C4	1.3810 (19)	C14—H14C	0.9800
C3—H3	0.9500	C15—C16	1.378 (2)
C4—C5	1.4892 (18)	C16—C17	1.3943 (19)
C5—H5A	0.9800	C16—H16	0.9500
C5—H5B	0.9800	C17—C18	1.4961 (19)
C5—H5C	0.9800	C18—H18A	0.9800
C6—C7	1.5159 (17)	C18—H18B	0.9800
C6—H6A	0.9900	C18—H18C	0.9800
			
N1—Zn1—N4	111.72 (4)	C8—C7—C12	119.12 (12)
N1—Zn1—Cl2	115.14 (3)	C8—C7—C6	118.15 (11)
N4—Zn1—Cl2	104.72 (3)	C12—C7—C6	122.74 (11)
N1—Zn1—Cl1	103.37 (3)	C9—C8—C7	121.27 (13)
N4—Zn1—Cl1	106.19 (3)	C9—C8—H8	119.4
Cl2—Zn1—Cl1	115.538 (13)	C7—C8—H8	119.4
C2—N1—N2	105.94 (10)	C10—C9—C8	119.63 (12)
C2—N1—Zn1	130.14 (9)	C10—C9—H9	120.2
N2—N1—Zn1	121.78 (8)	C8—C9—H9	120.2
C4—N2—N1	110.96 (10)	C9—C10—C11	119.51 (12)
C4—N2—C6	127.34 (11)	C9—C10—H10	120.2
N1—N2—C6	121.69 (10)	C11—C10—H10	120.2
C15—N3—N4	110.72 (11)	C10—C11—C12	121.65 (13)
C15—N3—C13	127.79 (11)	C10—C11—H11	119.2
N4—N3—C13	121.15 (10)	C12—C11—H11	119.2
C17—N4—N3	105.97 (10)	C11—C12—C7	118.78 (12)
C17—N4—Zn1	123.61 (9)	C11—C12—C13	118.43 (11)
N3—N4—Zn1	130.10 (8)	C7—C12—C13	122.78 (11)
C2—C1—H1A	109.5	N3—C13—C12	114.37 (10)
C2—C1—H1B	109.5	N3—C13—H13A	108.7
H1A—C1—H1B	109.5	C12—C13—H13A	108.7
C2—C1—H1C	109.5	N3—C13—H13B	108.7
H1A—C1—H1C	109.5	C12—C13—H13B	108.7
H1B—C1—H1C	109.5	H13A—C13—H13B	107.6
N1—C2—C3	109.73 (12)	C15—C14—H14A	109.5
N1—C2—C1	122.31 (12)	C15—C14—H14B	109.5
C3—C2—C1	127.95 (12)	H14A—C14—H14B	109.5
C4—C3—C2	106.51 (12)	C15—C14—H14C	109.5
C4—C3—H3	126.7	H14A—C14—H14C	109.5
C2—C3—H3	126.7	H14B—C14—H14C	109.5
N2—C4—C3	106.85 (11)	N3—C15—C16	106.88 (12)
N2—C4—C5	123.21 (12)	N3—C15—C14	122.67 (12)
C3—C4—C5	129.92 (13)	C16—C15—C14	130.42 (12)
C4—C5—H5A	109.5	C15—C16—C17	106.36 (12)
C4—C5—H5B	109.5	C15—C16—H16	126.8
H5A—C5—H5B	109.5	C17—C16—H16	126.8
C4—C5—H5C	109.5	N4—C17—C16	110.06 (12)
H5A—C5—H5C	109.5	N4—C17—C18	121.69 (12)
H5B—C5—H5C	109.5	C16—C17—C18	128.24 (12)
N2—C6—C7	113.37 (10)	C17—C18—H18A	109.5
N2—C6—H6A	108.9	C17—C18—H18B	109.5
C7—C6—H6A	108.9	H18A—C18—H18B	109.5
N2—C6—H6B	108.9	C17—C18—H18C	109.5
C7—C6—H6B	108.9	H18A—C18—H18C	109.5
H6A—C6—H6B	107.7	H18B—C18—H18C	109.5
			
N4—Zn1—N1—C2	144.03 (11)	C4—N2—C6—C7	−71.13 (16)
Cl2—Zn1—N1—C2	24.75 (12)	N1—N2—C6—C7	109.17 (12)
Cl1—Zn1—N1—C2	−102.19 (11)	N2—C6—C7—C8	109.89 (13)
N4—Zn1—N1—N2	−55.03 (10)	N2—C6—C7—C12	−70.62 (15)
Cl2—Zn1—N1—N2	−174.31 (8)	C12—C7—C8—C9	1.10 (19)
Cl1—Zn1—N1—N2	58.75 (9)	C6—C7—C8—C9	−179.40 (12)
C2—N1—N2—C4	0.12 (14)	C7—C8—C9—C10	0.6 (2)
Zn1—N1—N2—C4	−164.83 (9)	C8—C9—C10—C11	−1.0 (2)
C2—N1—N2—C6	179.86 (11)	C9—C10—C11—C12	−0.3 (2)
Zn1—N1—N2—C6	14.91 (15)	C10—C11—C12—C7	1.91 (19)
C15—N3—N4—C17	0.24 (14)	C10—C11—C12—C13	−179.28 (12)
C13—N3—N4—C17	174.00 (11)	C8—C7—C12—C11	−2.29 (18)
C15—N3—N4—Zn1	−173.30 (9)	C6—C7—C12—C11	178.22 (11)
C13—N3—N4—Zn1	0.46 (16)	C8—C7—C12—C13	178.96 (12)
N1—Zn1—N4—C17	170.83 (10)	C6—C7—C12—C13	−0.53 (19)
Cl2—Zn1—N4—C17	−63.89 (11)	C15—N3—C13—C12	−78.13 (16)
Cl1—Zn1—N4—C17	58.82 (11)	N4—N3—C13—C12	109.26 (13)
N1—Zn1—N4—N3	−16.63 (12)	C11—C12—C13—N3	105.38 (13)
Cl2—Zn1—N4—N3	108.65 (10)	C7—C12—C13—N3	−75.87 (15)
Cl1—Zn1—N4—N3	−128.64 (10)	N4—N3—C15—C16	−0.62 (14)
N2—N1—C2—C3	−0.15 (14)	C13—N3—C15—C16	−173.86 (12)
Zn1—N1—C2—C3	163.06 (9)	N4—N3—C15—C14	177.89 (12)
N2—N1—C2—C1	178.95 (12)	C13—N3—C15—C14	4.6 (2)
Zn1—N1—C2—C1	−17.83 (19)	N3—C15—C16—C17	0.73 (15)
N1—C2—C3—C4	0.13 (16)	C14—C15—C16—C17	−177.61 (14)
C1—C2—C3—C4	−178.91 (13)	N3—N4—C17—C16	0.24 (14)
N1—N2—C4—C3	−0.04 (15)	Zn1—N4—C17—C16	174.30 (9)
C6—N2—C4—C3	−179.76 (12)	N3—N4—C17—C18	−178.81 (12)
N1—N2—C4—C5	178.73 (12)	Zn1—N4—C17—C18	−4.75 (18)
C6—N2—C4—C5	−1.0 (2)	C15—C16—C17—N4	−0.61 (15)
C2—C3—C4—N2	−0.05 (15)	C15—C16—C17—C18	178.36 (14)
C2—C3—C4—C5	−178.72 (13)		
